# Chinese herbal medicine on treating obese women with polycystic ovary syndrome: A systematic review and meta-analysis protocol

**DOI:** 10.1097/MD.0000000000022982

**Published:** 2020-12-04

**Authors:** Ning Ding, Rensong Yue, Lizhen Wang, Hongjing Yang

**Affiliations:** a Hospital of Chengdu University of Traditional Chinese Medicine; b Chengdu University of Traditional Chinese Medicine, Chengdu, China.

**Keywords:** Chinese herbal medicine, meta-analysis, PCOS, protocol, systematic review

## Abstract

**Introduction:**

Known as an endocrine disorder, Polycystic ovary syndrome (PCOS) has posed an influence on 6% to 20% reproductive females worldwide. The commonly used pharmaceutical agents currently are Oral Contraceptives (OCs) and insulin-sensitizing agents. However, OCs is not appropriate for females pursuing pregnancy. Furthermore, some of insulin-sensitizing agents are found to be related to a high incidence of gastrointestinal adverse effects. In this regard, the effectiveness of Chinese herbal medicine in handling reproductive and metabolic defects simultaneously has been proved by extensive evidence. In this research, the effectiveness and safety of Chinese herbal medicine for obese females with PCOS were examined.

**Methods and analysis:**

In the systematic review, we searched databases of AMED, Science Online, EMbase, WorldSciNet, the Cochrane Library, PubMed, Nature, MEDLINE, China National Knowledge Infrastructure, the Wanfang Databse and China Biology Medicine Disc and the Chongqing VIP Chinese Science and Technology Periodical Database, to find out the papers published in Chinese or English by September 25, 2020 in this field. In addition, potential reference lists, relevant conference proceedings, qualified studies, related system reviews and other resources were also considered. Two researchers were responsible for independently selecting the research papers, collecting data, and evaluating research quality. Moreover, the data were synthesized with the combination of a fixed-effects or random-effects model with the heterogeneity test. According to the objective and self-reported assessment, the primary outcomes will be Nausea and vomiting were primary outcomes. RevMan 5 software was used to analyze the collected data, the evidence level of which was evaluated by GRADE. The selection between the fixed-effects and random-effects models was determined by the heterogeneity level. In addition to the 95% Confidence Interval (CI), odds ratio (OR), or risk ratio (RR) was applied to the 2 categories. Moreover, 95% CI and standardized mean difference (SMD) or the weighted mean difference (WMD) were taken as the continuous variables. When existing meaningful heterogeneity could not be explained by any assessment such as subgroup analysis, we would not conduct a meta-analysis. During the subgroup analysis, each subgroup in specific cases should be comprehensively considered.

**Ethics and dissemination:**

The evaluation of rights or personal information of patients was not involved in the systematic review. Hence, we need not gain approval from ethical institutions. This paper will be present at related conferences for communication and published in journals.

**Registration:**

Open Science Framework (OSF) Preregistration: osf.io/yp86h.

## 1 Introduction

Known as an endocrine disorder, polycystic ovary syndrome (PCOS) has influenced 6% to 20% reproductive females (depending on criteria and definitions).^[1]^ Its main symptoms include syndromes as oligo/anovulation, hyperandrogenism, hirsutism, obesity, and polycystic ovaries on ultrasonogram.^[2]^ The potential pathogenesis of PCOS is related to Insulin resistance (IR) and compensatory hyperinsulinemia.^[3]^ Existing pharmaceutical agents cover oral contraceptives (OCs)^[4,5]^ and insulin-sensitizing agents.^[1,6]^ However, OCs are not appropriate for women pursuing pregnancy; while the association of metformin, which aims to manage metabolic disorders, with a high incidence of gastrointestinal adverse effects has been reported.^[7]^

Traditional Chinese medicine (TCM) has the features of prevention of diseases occurrence and development, syndrome differentiation, and holistic conception. During the past centuries, it has gained plenty of interest from researchers due to its role in ameliorating IR^[8]^ as well as its benefits in the reproductivity of obese PCOS females.^[9]^ It was also found that Chinese herbal medicine was involved in treatments for almost 90% of females with newly diagnosed PCOS in Taiwan.^[10]^

To the best of our knowledge, the influence of Chinese herbal medicine on obese females with PCOS has not been studied yet. To make up this research gap, the present study was performed to examine the clinical safety and effectiveness of Chinese herbal medicine in the treatment of obese PCOS females.

## 2 Methods

### 2.1 Study registration

The protocol has been registered.

Registration: OSF Preregistration: osf.io/yp86 hours. The protocol was completed based on the guidelines of Preferred Reporting Items for Systematic Reviews and Meta-Analyses Protocols (PRISMAP). If it is required, any change in the full review will be reported.

### 2.2 Inclusion and exclusion criteria

#### 2.2.1 Inclusion criteria

Inclusion criteria: all randomized controlled trials (RCTs) with regard to Chinese herbal medicine therapy for the treatment of obese women with PCOS. Only papers in Chinese or English were enrolled. Study status, type or dates had no influence on the systematic review.

#### 2.2.2 Exclusion criteria

Exclusion criteria: animal studies, reviews, case series, quasi-RCTs, or Non-RCTs.

### 2.3 Types of participants

Obese female patients receiving Chinese herbal medicine treatment were invited without education or ethnicity restriction. Inclusion criteria of participants are listed as follows:

1. Females at the age of 15 to 40 years old;
2. Diagnosis of PCOS based on the modified Rotterdam criteria;
3. BMI ≥23 kg/m^2^;
4. Two years after menarche;
5. The homeostatic model assessment HOMA-IR was used to define IR: fasting insulin (μU/ml)×fasting glucose (mmol/L)/22.5. If the value is larger than 2.14, it is indicative of IR.^[11]^

### 2.4 Types of interventions

Experimental interventions were the dose-specific Chinese medicine preparation, the drug composition, or the combination of conventional treatment of Western Medicine with Chinese herbal medicine treatment. It included prescription and Chinese patent medicines, but not massage, acupuncture, intravenous medication, and other TCM treatments. In addition, the control group was set to merely accept western medicine, or the blank group was set to receive no treatment.

### 2.5 Types of outcome measures

#### 2.5.1 Primary outcomes

The comparison of the HOMA-IR baseline with the values after 3 months Chinese herbal medicine treatment was obtained as the primary outcome. In addition, insulin level and fasting glucose were determined.

#### 2.5.2 Secondary outcomes

1. Acne lesion counts, Ferriman-Gallwey score, blood pressure, BMI, waist/hip circumference, and weight.
2. Hormonal profile including follicle-stimulating hormone, luteinising hormone, dehydroepiandrosterone sulfate, sex hormone-binding globulin, testosterone, androstadienedione, and estradiol.
3. Adverse events.

## 3 Search methods for study identification

### 3.1 Electronic way

Relevant papers published as of September 25, 2020 were collected from AMED, Science Online, EMbase, WorldSciNet, the Cochrane Library, PubMed, Nature, MEDLINE, China National Knowledge Infrastructure, the Wanfang Databse and China Biology Medicine Disc and the Chongqing VIP Chinese Science and Technology Periodical Database.

### 3.2 Other search resources

**Figure 1:**
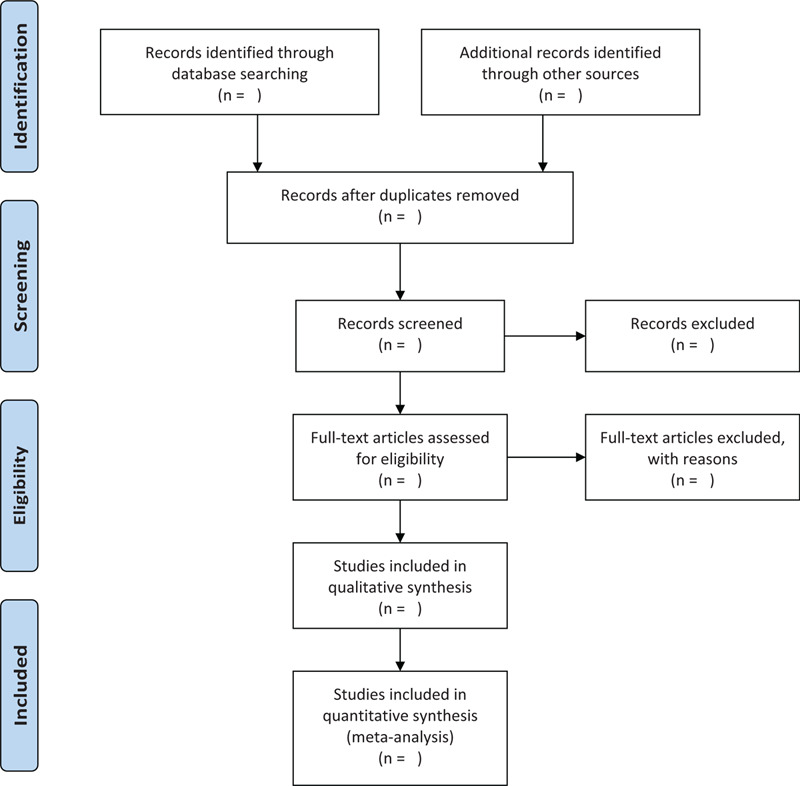
The research flowchart.

To determine other RCTs locations, we searched for relevant systematic reviews and a reference list of those qualified or potential literature. In addition, the authors were also contacted to obtain the latest clinical data for the convenience of ongoing RCTs. Notably, related conference proceedings were evaluated to identify the studies included in the review. Figure 1 shows the research flowchart.

### 3.3 Search strategy

Search terms consist of Chinese herbal medicine (like “traditional Chinese formula”, “traditional Chinese medicine”, “traditional Chinese prescription”) and obese women with PCOS (like “polycystic ovary syndrome”, “PCOS”, “obese”, “obesity”). Text words and MeSH were used. The detailed search strategy for the PubMed is available in the appendix.

Terms of “duonangluanchaozonghezheng”, “feipang”, “zhongyao”, “zhongcaoyao”, “caoyao”, “fangji” were used to search Chinese databases. The strategy was further modified to search other databases.

## 4 Data collection and analysis

### 4.1 Study selection

After the 2 researchers extracted information from the literature included in the study independently, they used it to generate a unified statistical table. First of all, they excluded duplicate records and ineligible studies, and then the full text of eligible studies was read to examine whether they met the inclusion criteria as described previously. For any disagreement between the 2 researchers, a third-party researcher would be invited to make the final judgement.

### 4.2 Data extraction and management

The data below were collected from each study: fund source and type, follow-up duration, primary outcomes, blinding method, allocation concealment method, intervention time, randomization, intervention groups sample size, year of publication, the first author, the reference ID, Chinese herbal medicine, age of the patient, control intervention type, and measure of outcome. If the reported data were insufficient, the author would be contacted. For any disagreement between the 2 researchers, a third-party researcher would be invited to make the final judgement.

### 4.3 Risk of bias assessment in included studies

Using the Cochrane collaboration risk-of-bias assessment, 2 researchers independently evaluated the quality of the literature reviewed and completed the STRICTA checklist.^[12]^ During the process, possible biases such as selective reporting, random sequence generation, incomplete outcome data, allocation concealment, blinding were considered. The risk of bias was divided into 3 levels from low, high to unclear based on the standards proposed in the Cochrane Intervention System Assessment Manual. Any discrepancy was solved by negotiation. If it failed, a third-party researcher would be invited to make the final judgement.

### 4.4 Treatment effect measures

ORs and SMD were adopted to measure the treatment effects with regard to dichotomous and continuous outcomes, respectively, which all reported 95% CIs.

### 4.5 Unit of analysis issues

The data of patients in RCTs were collected. In each treatment, individual multiple meta-analysis with over one Chinese herbal medicine group within an RCT was adopted. Data from the first sequence were applied to crossover studies. In addition, if many non-Chinese herbal medicine controls were included, all controls results were summed up to analyze the intervention and control groups.

### 4.6 Missing data management

The reason why data were lost during data screening and extraction were explored. The author of the study with missing data was contacted to get more information. If relevant data could not be found or get from the author, only available data were analyzed, followed by the explanation of the reason as well as the corresponding influence.

### 4.7 Heterogeneity assessment

A random- or fixed-effects model was built to perform a meta-analysis. As illustrated in the Cochrane Handbook for Systematic Reviews of Interventions, the heterogeneity can be evaluated by the Higgins*I*^2^ statistic and visually inspecting forest plot, a heterogeneity *x*^*2*^ test,.^[13,14]^ If the *P* value was over .10 and the *I*^2^ value was lower than 50%, a fixed-effects model was used to synthesize the data; otherwise, a random-effects model was adopted. If obvious heterogeneity were found from a set of studies, the causes of it, such as the variation degree in interventions and the characteristics of patients were discussed. If it was applicable, the subgroup analysis or the sensitivity group would be used to evaluate the heterogeneity level.

### 4.8 Reporting bias assessment

A funnel plot was adopted to assess the bias when more than 10 trials were included in the meta-analysis. The Egger and Begg tests were conducted to evaluate the asymmetry of the funnel plot, where significant publication bias is represented by the *P* value of less than .05.

### 4.9 Data synthesis

Data analysis was performed on the RevMan 5 software (V. 5.3; Copenhagen: The Nordic Cochrane Centre, The Cochrane Collaboration, 2014). Whether to use a random-effects model or a fixed-effects model was determined by the heterogeneity level. The index of WMD or SMD and 95% CI were continuous variables. The index of RR or OR and 95% CI were taken as 2 categorical variables. When existing meaningful heterogeneity could not be explained by any assessment such as subgroup analysis, we would not conduct a meta-analysis. During the subgroup analysis, each subgroup in specific cases should be comprehensively considered.

### 4.10 Subgroup analysis

In the subgroup analyses, the heterogeneity levels of the type of control (placebo or vehicle, or no medical treatment or conventional western medicine therapy), the type of Chinese herbal medicine (herbs composition, dosage), as well as the clinical difference, were taken into account.

### 4.11 Sensitivity analysis

A sensitivity analysis was conducted to testing whether review conclusions were robust, where the quality of heterogeneity, the sample size, and the statistic model (whether it is a random-effects model or a fixed-effects model) were considered.

### 4.12 Grading the evidence quality

The GRADE method was used to evaluate evidence quality for the obtained results.^[20]^ It involved publication risk of bias, estimate precision of effect, evidence directness, the heterogeneity, and risk of bias exhibited by studies. Evidence was classified into 4 levels, which are high risk, moderate risk, low risk, and very low risk.

### 4.13 Ethics and dissemination

The system review results will be presented at related conferences or published in journals reviewed by peers. Personal information of patients will be excluded by means of aggregated published data. Therefore, ethical approval or patients informed consent is unnecessary.

## 5 Discussion

PCOS is identified as a complex syndrome involving reproductive, endocrine, and metabolic disorder, which affects reproductive-age women worldwide and has predominant associations with infertility.^[15]^ The current treatments for PCOS focus on symptom management, such as treatment of obesity, irregular menses and hirsutisms. However, this clinical strategy is shortsighted and limited since increasing evidence links PCOS to a number of metabolic morbidities such as T2DM (type 2 diabetes mellites), obesity, metabolic syndrome, fatty liver disease, and endometrial cancer.^[11,16–18]^ Meanwhile, PCOS-related symptoms and complications may result in nonmetabolic morbidities such as mood disorders^[19–21]^ (depression, anxiety as examples), social and marital conflicts, thus leading to significant reduction in quality of life.^[22]^

Traditional Chinese medicine gains reputation by its holistic conception of disease treatment itself rather than only symptoms. A recent study concluded the potential role of Chinese herbal medicine in preventing T2DM-related complications among patients with PCOS.^[23]^ In another study, the compounds isolated from herbs were found effective in treating PCOS; moreover, both reproductive and metabolic defects could be targeted by the combination with a herbal formula.^[24]^

Chinese herbal medicine has broad application prospects for obese women with PCOS. In this paper, a systematic review and meta-analysis were performed to demonstrate the safety and effectiveness of Chinese herbal medicine in the treatment of obese women with PCOS. In addition, the review result may provide a reference for health policy makers, practitioners, and patients.

## Author contributions

**Conceptualization:** Ning Ding, Rensong Yue, Hongjing Yang.

**Data curation:** Ning Ding, Rensong Yue, Hongjing Yang.

**Formal analysis:** Lizhen Wang.

**Funding acquisition:** Ning Ding, Rensong Yue.

**Methodology:** Lizhen Wang, Hongjing Yang.

**Project administration:** Ning Ding, Rensong Yue, Hongjing Yang.

**Writing – original draft:** Ning Ding, Hongjing Yang.

**Writing – review & editing:** Rensong Yue.
